# Integra® Dermal Regeneration Template: From Design to Clinical Use

**DOI:** 10.7759/cureus.38608

**Published:** 2023-05-05

**Authors:** Philippe Taupin, Ankur Gandhi, Sunil Saini

**Affiliations:** 1 Medical Affairs, Integra LifeSciences, Princeton, USA; 2 Research and Development, Integra LifeSciences, Princeton, USA

**Keywords:** wound reconstruction, trauma, integra, dermal regeneration template, collagen, burn

## Abstract

Integra^®^ Dermal Regeneration Template (IDRT, Integra LifeSciences, Princeton, NJ, USA) is a bilayer membrane developed, by Yannas and Burke in the 1980s, to fulfill the unmet need of surgeons having a readily available off-the-shelf dermal regeneration method. IDRT is composed of a sheet of porous cross-linked type I collagen and glycosaminoglycans, with a semi-permeable silicone sheet cover. IDRT is bio-engineered, from adult bovine Achilles tendons and chondroitin-6-sulfate derived from shark cartilage, in a multi-step process involving cross-linking using glutaraldehyde. By design, the composition, porosity, and biodegradation rate of IDRT guides the mechanism of wound repair towards a regenerative pathway. Its mechanism of action involves four distinct phases: imbibition, fibroblast migration, neovascularization, and remodeling/maturation. Originally developed for the post-excisional treatment of deep-partial to full-thickness burns where autograft is limited, over the years its use has expanded to reconstructive surgery.

## Introduction and background

Integra® Dermal Regeneration Template (IDRT, Integra LifeSciences, Princeton, NJ, USA) was developed, by Yannas and Burke in the 1980s, to fulfill the unmet need of surgeons having a readily available off-the-shelf dermal regeneration method [1-3]. IDRT is a bilayer membrane composed of a collagen/glycosaminoglycan (GAG) matrix and a silicone layer. It is designed to provide a solution for immediate coverage and closure of the wounds and functions jointly with a split-thickness skin graft (STSG) to regenerate a functional dermis [4]. IDRT minimizes the risk of contracture and scar formation typically associated with skin grafting [5]. It provides an alternative solution to skin grafting and flap reconstruction, which offer immediate coverage but are limited by their supply, inability to cover exposed structures, and donor-site morbidities [6,7]. IDRT was initially developed as a dermal scaffold for use in full-thickness thermal injuries, particularly in cases with insufficient tissue available for donor sites from which autograft can be harvested [8]. In this manuscript, we will review the design properties of IDRT that guide the mechanism of wound repair towards a regenerative pathway.

## Review

Design

Yannas and Burke published the design, composition, and control of IDRT [1-3]. Three physio-chemical properties are designed in IDRT to induce dermal regeneration: composition, porosity, and biodegradation rate.

Composition: wound repair, regenerative pathway, and wound covering

IDRT is composed of type I collagen, GAG, and a silicone layer. The collagen used in IDRT is derived from adult bovine Achilles tendons. Type I collagen is the most abundant type of collagen and protein in the human body and is the major component of the extracellular matrix [9]. Collagen fibers are synthesized primarily by fibroblasts in the deeper layers of the skin. Collagen has a low immunogenicity and antigenicity response [10]. The mechanical properties of collagen and its biodegradation rate can be controlled through cross-linking, allowing it to be optimized for specific applications and indications [1]. Collagen has the possibility of being hydrated, which provides a flush interface between the template and the wound, preventing air pockets that might cause the area to be susceptible to bacterial proliferation [1].

GAGs are long linear polysaccharides consisting of repeating disaccharide units, and specifically, chondroitin-6-sulfate (C6S) is used in IDRT [2]. GAGs are weak antigens and block platelets from aggregating [11]. This decrease in platelet aggregation limits the immune response during wound repair by reducing inflammation and the release of cytokines, particularly transforming growth factor beta (TGF-β) [12]. GAGs stimulate the formation and stabilization of collagen fibrils and fibers of various lengths and diameters, as well as control the spatial orientation of the fibers [13]. To effectively immobilize the GAG to the collagen, the complex collagen-GAG is cross-linked using glutaraldehyde [1]. Incorporating GAGs into the matrix increases the modulus of elasticity, generates a matrix with a more dilated porosity and contributes to the control of the biodegradation rate of the cross-linked collagen-GAG matrix [1].

The collagen/GAG matrix acts as the dermal replacement layer. It has a thickness of approximately 1 mm by 0.8 mm. Both type I collagen and C6S are biodegradable and non-toxic. Clinically, the collagen/GAG complex contributes more to the regenerative process than just providing a template that biodegrades and is non-toxic. The complex inhibits platelet aggregation, which is the first step in the wound repair mechanism [11]. It minimizes the immune response, reducing inflammation and thus reducing the release of TGF-β, which is involved in fibroblast differentiation leading to wound contraction [12]. This delays the wound repair process, allowing for the ingrowth of a neodermis in the dermal matrix, by fibroblasts and endothelial cells [14]. Hence, the initial reduction in the inflammatory response by IDRT guides the mechanism of wound repair towards a regenerative pathway rather than scar formation.

The silicone (polysiloxane polymer) layer acts as a temporary epidermal substitute. It has a thickness of 0.23 mm. The silicone layer prevents fluid egress, functions to control heat and moisture loss, and provides a protective barrier from infection [14]. If the water flux rate through the silicone layer is too high, it can lead to early dehydration of the template. If it is too low, water will accumulate and subsequently cause edema [1]. The silicone layer in IDRT was designed to provide a water flux that is the same as that across the normal epidermis at 0.5 mL/cm^2^/h [15]. The silicone layer provides a flexible adherent covering of the wound, increases the tear strength of IDRT, and delays the need for an autograft. It is temporary, as it is removed once a neodermis has formed and replaced with an epidermal autograft (STSG) [4].

Porosity: cell infiltration and proliferation, and fibroblasts migration

The porosity of dermal matrices affects the ability of fibroblasts to proliferate, differentiate, and produce collagen [16]. The absence of pores or a slow biodegradation rate will result in the early formation of a dense fibrous tissue capsule at the dermal matrix-wound bed interface and the eventual curtailment of cell migration [3]. The porosity of IDRT has been optimized for cell infiltration and proliferation and for nutrient access to support neodermis formation [3]. Incorporating GAGs in IDRT generates a matrix with a more dilated porosity; it has a pore size of 70 mm to 200 mm [3]. Due to the random pore structure, the fibroblasts migrate in random planes. Myofibroblasts subsequently do not properly align, suppressing wound contraction [3,14]. IDRT’s porosity delays contraction to allow for the growth of a neodermis by host fibroblasts and endothelial cells [4].

Biodegradation rate: inflammation and contraction

The biodegradation rate of IDRT is critical for neodermal regeneration. A rapid biodegradation rate results in an almost immediate loss of pore structure, making it ineffective for neodermal formation. A slow biodegradation rate would result in the formation of relatively dense fibrotic tissue between the silicone layer and the wound bed [1]. The biodegradation rate of IDRT has been engineered to reduce inflammation and contraction at a cellular level, e.g., the GAGs inhibit platelet aggregation leading to a reduction in inflammation and thus contraction by myofibroblasts, and to support the infiltration and proliferation of fibroblasts, endothelial cells, smooth muscle cells, and keratinocytes in the matrix [12,14]. The IDRT collagen-GAG matrix is mostly degraded 30 days after application, with small fragments observed up to 12 months after placement [17,18].

Mechanism of action

Four distinct phases of dermal regeneration have been reported histologically by evaluating punch biopsies from patients undergoing reconstructive surgery using IDRT, for the release of contracture followed by resurfacing of tight or painful scars [19]. The four distinct phases of dermal regeneration using IDRT are imbibition, fibroblast migration, neovascularization, and remodeling and maturation.

Imbibition is observed within minutes of applying IDRT, with exudate filling the interstices of the matrix [19]. The exudate contains red blood cells and fibrin, providing certain cytokines for the wound's regenerative process. The fibrin fosters adherence of the matrix to the wound, similarly to the adherence of skin grafting. In this phase, the matrix swells over the first few days, and inflammatory cells become present in the matrix at 7-10 days [17]. During this phase, the levels of TGF-β and cytokines begin to rise but eventually diminish due to the GAG content of the IDRT blocking platelets from aggregating [12]. This lower concentration of cytokines causes a lower level of immune cell infiltration and consequently minimal inflammation within the matrix during this phase. The IDRT properties have low immunogenicity and low antigenicity, further reducing the immune response and inflammation [20]. During this phase, the matrix changes in coloration from red to pale yellow by week two after matrix placement, with some red patches remaining where there is a high concentration of red blood cells.

Fibroblast migration is observed by day seven after placement of the dermal matrix, with myofibroblasts located at the base of the matrix. Fibroblasts use the collagen fibers of the dermal matrix as a scaffold and, by the third week, settle along the interstices of the matrix and start producing host collagen [19]. The reduction in TGF-β levels and low inflammatory response down-regulate the differentiation of fibroblasts into myofibroblasts, the promoters of wound contraction [12]. During this process, fibroblasts are unable to migrate in a plane parallel to the epithelium, but rather migrate in random patterns due to the engineered pore structure of IDRT. As a result, fibroblasts that differentiate into myofibroblasts cannot contract efficiently because they are unable to interact with one another and are positioned in random planes. All of which contribute to a delay in wound contraction, a key factor towards the regenerative pathway. Since the fibroblasts and myofibroblasts are in random positions, the collagen is deposited in random orientations, unlike in wound dermal repair, where collagen deposition is parallel to the wound bed [1].

Neovascularization, contributing to neodermal regeneration, starts at the end of the second week with endothelial cell migration within the collagen-GAG matrix [19]. These cells form solid columns that stain positively for the endothelial markers CD31 and CD34. The formation of vascular channels begins 14-17 days after application, and lumen formation begins during the third week [17,21]. Neovascularization is established by the fourth week, by which time the silicone layer is removed and a skin graft (STSG) is applied onto the neodermis [4]. During this phase, the matrix changes in coloration from pale yellow to peach; an indicator that the neodermis is fully vascularized.

The remodeling and maturation start when the dermal matrix has been populated with fibroblasts and the interstices are full of new host collagen that gradually replaces the IDRT collagen [19]. The fibroblasts that are present in the wound deposit collagen into the matrix, which is indistinguishable histologically from normal dermal collagen, with no resemblance to hypertrophic scar tissue or keloid. The random pore structure allows for the collagen to be deposited in random planes. Early in this phase, the neodermis becomes thicker than the normal dermis, but as the neodermis matures over the subsequent months, it becomes thinner and more pliable [22]. The IDRT collagen is mostly degraded 30 days after application [17,19]. The delay in wound contraction permits the remodeling and maturation phases to occur and a neodermis to form, as opposed to a scar [4,5]. During this stage, the epidermal autograft (STSG), placed after the removal of the silicone layer, becomes adherent to the surface of the neodermis, and the dermal-epidermal junction develops rete ridges [19]. Rete ridges are characteristic of basement membrane maturation. IDRT assists in basal lamina formation by inhibiting the degradation of newly formed GAG, which develops into the basal lamina of the basement membrane [23]. This completes the regeneration of the skin, providing all three main structures: basement membrane, epidermis, and dermis. The regenerated skin is mechanically competent, vascularized, and sensitive to touch and temperature but lacks certain appendages such as hair follicles and sweat glands [4,5,19]. No adnexal structures, nerve endings, or elastic fibers are present [19].

Defining the four phases of dermal regeneration using IDRT allows correlation between the extent of vascularization of the matrix with the clinical appearance of the matrix. The change in color of the matrix is a predictor of its vascularization. When the color has progressed from red through pale yellow and finally to peach, the neodermis is fully vascularized [22].

Clinical use

In the US, IDRT was approved in March 1996 by the Food and Drug Administration for the treatment of life-threatening burn injuries and reconstruction of scar contractures. Integra® Bilayer Wound Matrix (IBWM), a bilayer matrix composed of the same collagen and GAG matrix based on Integra dermal regeneration technology, received 510(k) clearance by the Food and Drug Administration in 2002 for the management of wounds; including partial and full-thickness wounds, pressure ulcers, venous ulcers, diabetic ulcers, chronic vascular ulcers, surgical wounds, trauma wounds, and draining wounds. Meshed variants of IDRT, Integra® Meshed Dermal Regeneration Template, and IBWM, Integra® Meshed Bilayer Wound Matrix (IMBWM), are available.

The use of Integra bilayer matrices for wound management is a two-step process; the placement of the dermal matrix on top of a debrided wound bed, followed by the application of a STSG [4]. An interval period of three to four weeks is usually required between the two steps to allow ample time for the fibroblasts to proliferate and neovascularization to occur [20]. The wound is ready for skin grafting (STSG) once the color underneath the silicone layer changes from tan to pink or peach [22]. The superficial silicone layer is then removed in the operating room, and the skin graft is placed [4].

The use of IDRT has been evaluated in multiple clinical trials and studies, including randomized controlled trials and prospective clinical trials, for the treatment of life-threatening burn injuries and the reconstruction of scar contractures [24-28]. The safety and effectiveness of IDRT were evaluated in a post-approval study involving 216 burn injury patients, with a mean total body surface area burned of 36.5%. In this study, the authors reported a mean take rate of IDRT at all burn wound sites of 76.2% (median take of 98%) and a mean take rate for STSG of 87.7% (median take of 95%) [8]. The successful use of IBWM has been reported for the management of complex lower extremity soft-tissue reconstruction, traumatic degloving injuries, purpura fulminans, complex highly contaminated combat-related injuries, hand wounds associated with exposed bone, full-thickness scalp and calvarial defect with exposed dura, joints and/or tendons from trauma or cancer resection, and for complex Mohs defects [29-40]. Shakir et al. reported, in a retrospective case-control study of 147 patients undergoing lower extremity wound reconstruction using IBWM, a 70% rate of wound healed at 180 days post-surgery, with reconstructive failure associated with tendon exposure, bone exposure, and bone excision [41]. In a five-year retrospective review comparing the use of IMBWM followed by STSG combined with negative pressure wound therapy (NPWT) versus IMBWM followed by STSG alone for the management of complex extremity wounds, the authors reported a significantly greater take of IMBWM+STSG combined with NPWT (96.8%) compared to without NPWT (85.1%, p=0.03). In this study, there were significantly fewer reapplications of IMBWM required when combined with NPWT versus without NPWT (3.2% vs 14.9%, p=0.03). There were significantly fewer postoperative complications (exposed fascia, exposed tendon, infection), prior to STSG when IMBWM was combined with NPWT versus without NPWT (3.2% vs 14.9%, p=0.03) [42].

Despite this clinical success, the use of Integra bilayer matrices is not without its limitations. IDRT and IBWM are intended to be utilized in a two-stage procedure, requiring two surgeries and multiple dressing changes. Two-stage procedures are generally conducted on patients with large defects who would benefit from a secondary skin graft. Single-stage procedures using these bilayer dermal matrices have been reported [38,43,44]. Particularly, single-stage procedures through epithelialization, via secondary intention, may offer the opportunity to avoid multiple operations while requiring frequent dressing changes, and reduce donor-site morbidity and the reliance on patient compliance [38,43]. An additional consideration for these bilayer dermal matrices is their associated financial costs. These costs, however, must be balanced against the associated costs and comorbidity of autologous reconstruction. The use of tissue flaps or tissue expansion is also costly, requires multiple procedures, and the need for frequent office visits. Kozak et al. (2020) reported in a large, multi-institutional study comparing three treatment modalities for complex lower extremity reconstruction that free flap reconstruction leads to a longer hospital length of stay, increased numbers of readmissions and reoperations, and high costs; local autologous tissue rearrangement provides coverage at minimal costs and decreased readmissions and reoperations; IBWM can be effectively used in certain patient populations, such as older, obese patients without exposed bone while reducing costs and decreasing hospital length of stay [45]. Hence, the associated costs of an autologous reconstruction must be balanced against the distinct benefits of using IDRT and IBWM and compared with current alternative methodologies and the clinical outcomes sought [45].

## Conclusions

IDRT is a readily available off-the-shelf dermal regeneration method, and an alternative solution to skin grafting and flap reconstruction that are limited by their supply, inability to cover exposed structures, and donor-site morbidities. By design, IDRT provides a base for revascularization and neodermal formation and uses a regenerative pathway to achieve wound closure. In a two-stage procedure, IDRT leads to favorable cosmesis and functional outcomes. IDRT, IBWM, and their variants have been used successfully for the management of acute and chronic wounds and have become part of the armamentarium of the burn surgeon for acute burns, burn reconstruction, and reconstructive surgery.

## References

[1] Yannas IV, Burke JF (1980). Design of an artificial skin. I. Basic design principles. J Biomed Mater Res.

[2] Yannas IV, Burke JF, Gordon PL, Huang C, Rubenstein RH (1980). Design of an artificial skin. II. Control of chemical composition. J Biomed Mater Res.

[3] Dagalakis N, Flink J, Stasikelis P, Burke JF, Yannas IV (1980). Design of an artificial skin. Part III. Control of pore structure. J Biomed Mater Res.

[4] Yannas IV, Burke JF, Orgill DP, Skrabut EM (1982). Wound tissue can utilize a polymeric template to synthesize a functional extension of skin. Science.

[5] Burke JF, Yannas IV, Quinby WC Jr, Bondoc CC, Jung WK (1981). Successful use of a physiologically acceptable artificial skin in the treatment of extensive burn injury. Ann Surg.

[6] Fischer JP, Sieber B, Nelson JA (2013). A 15-year experience of complex scalp reconstruction using free tissue transfer-analysis of risk factors for complications. J Reconstr Microsurg.

[7] Jordan DJ, Malahias M, Hindocha S, Juma A (2014). Flap decisions and options in soft tissue coverage of the lower limb. Open Orthop J.

[8] Heimbach D, Luterman A, Burke J (1988). Artificial dermis for major burns. A multi-center randomized clinical trial. Ann Surg.

[9] Davison-Kotler E, Marshall WS, García-Gareta E (2019). Sources of collagen for biomaterials in skin wound healing. Bioengineering (Basel).

[10] Geanaliu-Nicolae RE, Andronescu E (2020). Blended natural support materials-collagen based hydrogels used in biomedicine. Materials (Basel).

[11] Luzzatto G, Paolini R, Stevanato F, Simioni P, Cella G (1989). Effects of glycosaminoglycans and protamine chloridrate on platelet aggregation induced by collagen and thrombin. Angiology.

[12] Boo S, Dagnino L (2013). Integrins as modulators of transforming growth factor beta signaling in dermal fibroblasts during skin regeneration after injury. Adv Wound Care.

[13] Sadowska M, Gutowska J, Malesa M (2005). Effect of glycosaminoglycans on reconstitution of collagen fibrils. Pol J Food Nutr Sci.

[14] Yannas IV, Orgill DP, Burke JF (2011). Template for skin regeneration. Plast Reconstr Surg.

[15] Kamolz LP, Lumenta DB (2013). Dermal Replacements in General, Burn, and Plastic Surgery. Dermal Replacements in General, Burn, and Plastic Surgery.

[16] Boekema BK, Vlig M, Olde Damink L, Middelkoop E, Eummelen L, Bühren AV, Ulrich MM (2014). Effect of pore size and cross-linking of a novel collagen-elastin dermal substitute on wound healing. J Mater Sci Mater Med.

[17] Stern R, McPherson M, Longaker MT (1990). Histologic study of artificial skin used in the treatment of full-thickness thermal injury. J Burn Care Rehabil.

[18] Vana LP, Battlehner CN, Ferreira MA, Caldini EG, Gemperli R, Alonso N (2020). Comparative long-term study between two dermal regeneration templates for the reconstruction of burn scar contractures in humans: clinical and histological results. Burns.

[19] Moiemen NS, Staiano JJ, Ojeh NO, Thway Y, Frame JD (2001). Reconstructive surgery with a dermal regeneration template: clinical and histologic study. Plast Reconstr Surg.

[20] Michaeli D, McPherson M (1990). Immunologic study of artificial skin used in the treatment of thermal injuries. J Burn Care Rehabil.

[21] Campitiello E, Della Corte A, Fattopace A, D'Acunzi D, Canonico S (2005). The use of artificial dermis in the treatment of chronic and acute wounds: regeneration of dermis and wound healing. Acta Biomed.

[22] Moiemen NS, Vlachou E, Staiano JJ, Thawy Y, Frame JD (2006). Reconstructive surgery with Integra dermal regeneration template: histologic study, clinical evaluation, and current practice. Plast Reconstr Surg.

[23] David G, Bernfield MR (1979). Collagen reduces glycosaminoglycan degradation by cultured mammary epithelial cells: possible mechanism for basal lamina formation. Proc Natl Acad Sci U S A.

[24] Lorenz C, Petracic A, Hohl HP, Wessel L, Waag KL (1997). Early wound closure and early reconstruction. Experience with a dermal substitute in a child with 60 per cent surface area burn. Burns.

[25] Hunt JA, Moisidis E, Haertsch P (2000). Initial experience of Integra in the treatment of post-burn anterior cervical neck contracture. Br J Plast Surg.

[26] Heimbach DM, Warden GD, Luterman A (2003). Multicenter postapproval clinical trial of Integra® dermal regeneration template for burn treatment. J Burn Care Rehabil.

[27] Wisser D, Rennekampff HO, Schaller HE (2004). Skin assessment of burn wounds covered with a collagen based dermal substitute in a 2 year-follow-up. Burns.

[28] Moiemen N, Yarrow J, Hodgson E (2011). Long-term clinical and histological analysis of Integra dermal regeneration template. Plast Reconstr Surg.

[29] Helgeson MD, Potter BK, Evans KN, Shawen SB (2007). Bioartificial dermal substitute: a preliminary report on its use for the management of complex combat-related soft tissue wounds. J Orthop Trauma.

[30] Katrana F, Kostopoulos E, Delia G, Lunel GG, Casoli V (2008). Reanimation of thumb extension after upper extremity degloving injury treated with Integra®. J Hand Surg.

[31] Khashab ME, Rhee ST, Pierce SD, Khashab YE, Nejat F, Fried A (2009). Management of large scalp and skull defects in a severe case of Adams-Oliver syndrome. J Neurosurg Pediatr.

[32] Abbas Khan MA, Chipp E, Hardwicke J, Srinivasan K, Shaw S, Rayatt S (2010). The use of Dermal Regeneration Template (Integra®) for reconstruction of a large full-thickness scalp and calvarial defect with exposed dura. J Plast Reconstr Aesthet Surg.

[33] Corradino B, Di Lorenzo S, Leto Barone AA, Maresi E, Moschella F (2010). Reconstruction of full thickness scalp defects after tumour excision in elderly patients: our experience with Integra® dermal regeneration template. J Plast Reconstr Aesthet Surg.

[34] Khan MA, Ali SN, Farid M, Pancholi M, Rayatt S, Yap LH (2010). Use of dermal regeneration template (Integra) for reconstruction of full-thickness complex oncologic scalp defects. J Craniofac Surg.

[35] Smock ED, Barabas AG, Geh JL (2010). Reconstruction of a thumb defect with Integra following wide local excision of a subungual melanoma. J Plast Reconstr Aesthet Surg.

[36] Weigert R, Choughri H, Casoli V (2011). Management of severe hand wounds with Integra® dermal regeneration template. J Hand Surg.

[37] Shah A, Taupin P (2022). Strategies for extremity reconstruction with exposed bones and tendons using acellular dermal matrices: concept of sequential vascularization. Case Rep Plast Surg Hand Surg.

[38] Shah A, Taupin P (2022). Single-stage extremity reconstruction through the use of dermal matrices: the power of Integra(®) bilayer wound matrix in the face of medical comorbidities, patient preference and non-compliance. Case Rep Plast Surg Hand Surg.

[39] Prezzavento GE, Calvi RN, Rodriguez JA, Taupin P (2022). Integra dermal regeneration template in reconstructive surgery for cutaneous tumours: a two-year retrospective review. J Wound Care.

[40] Silk JA, Taupin P (2023). Serial application of meshed collagen-chondroitin silicone bilayer matrix to obtain full coverage over bone and tendon in challenging situations and medically compromised patients: a small case series. Wounds.

[41] Shakir S, Messa CA 4th, Broach RB (2020). Indications and limitations of bilayer wound matrix-based lower extremity reconstruction: a multidisciplinary case-control study of 191 wounds. Plast Reconstr Surg.

[42] Gonzalez GA, Castagno C, Carter J, Chellappan B, Taupin P (2022). Negative pressure wound therapy on complex extremity wounds requiring coverage with a meshed bilayer wound matrix: a retrospective analysis. J Wound Care.

[43] Burd A, Wong PS (2010). One-stage Integra reconstruction in head and neck defects. J Plast Reconstr Aesthetic Surg.

[44] Gabriel A, Wong W, Gupta S (2012). ﻿﻿﻿﻿﻿﻿﻿Single-stage reconstruction for soft tissue defects: a case series. Ostomy Wound Manage.

[45] Kozak GM, Hsu JY, Broach RB (2020). Comparative effectiveness analysis of complex lower extremity reconstruction: outcomes and costs for biologically based, local tissue rearrangement, and free flap reconstruction. Plast Reconstr Surg.

